# Confederate States Hospital Reports

**Published:** 1864-02

**Authors:** 


### CONFEDERATE STATES HOSPITAL REPORTS.

I.—*Report of Cases of Compound Comminuted Fracture of Femur, Chimborazo Hospital, Third Division.*

Surgeon E. H. SMITH, in charge.

Case 1.—H. C. Stevenson, company "K," Palmetto Sharp-Shooters; wounded 27th June, 1862; admitted 29th June, 1862.

Operation.—Circular amputation of "middle-third" of thigh, June 27th; having received compound comminuted fracture of femur; great laceration of the soft parts; furloughed 4th September.

Case 2.—W. L. Williamson, company "I," 28th Virginia; wounded 27th June, 1862; admitted 28th June, 1862.

Case.—No amputation was performed; but case progressed favorably and was furloughed 30th July, 1862.

Case 3.—C. Brett, company "B," 11th Alabama; wounded 27th June, 1862; admitted 2d July, 1863.

Operation.—Circular amputation of middle-third of thigh, June 28th, having received compound comminuted fracture of femur in lower-third; was transferred to Alabama hospital, July 15th.

Case 4.—J. K. P. Powell, company "C," 31st Georgia; wounded 26th June, 1862; admitted June 30th.

Operation.—Circular amputation of thigh in "middle-third," having received a compound comminuted fracture of knee-joint; was furloughed 26th August.

Case 5.—J. W. Cole, company "G," 28th North Carolina; wounded 27th June, 1862; admitted July 2d.

Operation.—June 28.—Circular amputation of thigh in lower-third, having received a compound comminuted fracture of bones of left leg near knee; was furloughed 19th July.

Case 6.—T. R. Howell, company "C," 4th Alabama; wounded 27th June, 1862; admitted 29th June.

Operation.—Circular amputation of thigh in lower-third, July 2d, having received a compound comminuted fracture of leg, left-tibia; died 4th July, of tetanus.

Case 7.—James A. Gibson, company "F," 7th Virginia; wounded 27th June, 1862; admitted July 1st.

Operation.—Circular amputation of thigh in lower-third, July 3d, having received compound comminuted fracture of knee-joint; was transferred and since *died* in private quarters.

Case 8.—R. Henderson, company "A," 21st North Carolina; wounded 3d May, 1862; admitted ——.

Case.—The ball passed directly through the knee-joint, fracturing the articulating surface of femur with tibia. This case remained under treatment in this hospital for four months, when he was thought sufficiently recovered to be furloughed. He, of course, had an anchylosed joint

Case 9.—Q. C. Haley, company "K," 38th North Carolina; wounded 2d July, 1863; admitted 16th July.

Case.—Ball passed through left thigh, fracturing bone at the lower-third. Furloughed September, 1863.

Case 10.—M. R. Brooks, company "F," 53d Virginia; wounded 3d July, 1863; admitted 28th September.

Case.—Ball passed through thigh, fracturing bone at lower-third. When he was furloughed he had no use of the leg, and, to all appearance, anchylosis of the knee-joint had taken place.

Case 11.—F. Williams, company "A," 46th North Carolina; wounded December 13, 1862; admitted December 24th.

Case.—Ball passed through thigh, fracturing upper-third of femur. He was treated with Smith's anterior splint, but died March 27th, 1863.

Case 12.—W. H. Harrison, company "A," 41st Virginia; admitted June 1st, 1862.

Case.—Compound comminuted fracture of femur, ball passing through trochanter major. Died 23d June, 1862.

Case 13.—J. W. Colt, company "K," 5th Texas; wounded June, 1862.

Case.—Ball passed through thigh, fracturing upper-third of femur. He was heated with Smith's anterior splint. Furloughed Sept., 1862.

SUMMARY.

| | |
|---|---|
| Cases, | 13 |
| Amputations, | 6 |
| Conservative treatment, | 7 |
| Knee-joint, | 3 |

| | |
|---|---|
| Thigh, | 10 |
| Anchylosis of knee-joint, | 2 |
| Furloughed, | 8 |
| Transferred, | 3 |
| Died, | 4 |
| Amputations, 6—Deaths, | 2 |
| Conservative surgery, 7—Deaths, | 2 |

All of the aforenamed patients who have been *furloughed* were transferred to their homes when their speedy recovery was manifest.

---

II.—*Synopsis of the Consolidated Reports of the Hospitals in the Department of Virginia, from September*, 1862, *to December*, 1863, *inclusive*. By Surgeon W. A. CARRINGTON, Medical Director.

| | |
|---|---|
| Total number admitted | 293,165 |
| " " transferred | 127,530 |
| " " returned to duty | 98,340* |
| " " furloughed | 39,665 |
| " " discharged | 4,441† |
| " " deserted | 4,446 |
| " " died | 10,248 |
| " " in hospital, January 1, 1864 | 8,495 |

* From the total returned to duty, must be deducted 2,465 prisoners returned to quarters—leaving 95,875.

† From the total discharged the service, must be deducted 1,634 prisoners discharged and sent home on parole—leaving 2,807.

Adding up the number accounted for, (returned to duty, furloughed, died, deserted, discharged and on hand,) and adding 9,134, the number transferred to hospitals *out of the State*, we have the true number of cases treated in the Virginia hospitals from September, 1862, to December, 1863, inclusive, 174,767—giving the grand ratio of mortality, 5.86 per centum.

The largest number under treatment at any one time was in January, 1863—18.876.

The smallest population occurred in October, 1863—7.841.

